# Can feedback approaches reduce unwarranted clinical variation? A systematic rapid evidence synthesis

**DOI:** 10.1186/s12913-019-4860-0

**Published:** 2020-01-16

**Authors:** Reema Harrison, Reece Amr Hinchcliff, Elizabeth Manias, Steven Mears, David Heslop, Victoria Walton, Ru Kwedza

**Affiliations:** 1 School of Public Health and Community Medicine, University of New South Wales, Samuels Building (f25), Sydney, NSW 2052 Australia; 2 School of Public Health and Social Work, Queensland University of Technology, Brisbane, QLD 4059 Australia; 3 Melbourne School of Health Sciences, The University of Melbourne, Melbourne, Australia; 4 School of Nursing and Midwifery, Deakin University, Geelong, Australia; 5 Information Specialist, Hunter New England Medical Library, New Lambton, NSW 2350 Australia; 6 Cancer Institute New South Wales, Level 9, 8 Central Avenue, Australian Technology Park, Eveleigh NSW 2015, PO Box 41, Alexandria, NSW 1435 Australia; 7 Centre for Rural Health-North Coast, School of Rural Health, University of Sydney, Lismore, New South Wales Australia

**Keywords:** Unwarranted clinical variation, Clinical variation, Health services, Facilitated feedback, Clinician feedback, Effective care

## Abstract

**Background:**

Assessment of clinical variation has attracted increasing interest in health systems internationally due to growing awareness about better value and appropriate health care as a mechanism for enhancing efficient, effective and timely care. Feedback using administrative databases to provide benchmarking data has been utilised in several countries to explore clinical care variation and to enhance guideline adherent care. Whilst methods for detecting variation are well-established, methods for determining variation that is unwarranted and addressing this are strongly debated. This study aimed to synthesize published evidence of the use of feedback approaches to address unwarranted clinical variation (UCV).

**Methods:**

A rapid review and narrative evidence synthesis was undertaken as a policy-focused review to understand how feedback approaches have been applied to address UCV specifically. Key words, synonyms and subject headings were used to search the major electronic databases Medline and PubMed between 2000 and 2018. Titles and abstracts of publications were screened by two reviewers and independently checked by a third reviewer. Full text articles were screened against the eligibility criteria. Key findings were extracted and integrated in a narrative synthesis.

**Results:**

Feedback approaches that occurred over a duration of 1 month to 9 years to address clinical variation emerged from 27 publications with quantitative (20), theoretical/conceptual/descriptive work (4) and mixed or multi-method studies (3). Approaches ranged from presenting evidence to individuals, teams and organisations, to providing facilitated tailored feedback supported by a process of ongoing dialogue to enable change. Feedback approaches identified primarily focused on changing clinician decision-making and behaviour. Providing feedback to clinicians was identified, in a range of a settings, as associated with changes in variation such as reducing overuse of tests and treatments, reducing variations in optimal patient clinical outcomes and increasing guideline or protocol adherence.

**Conclusions:**

The review findings suggest value in the use of feedback approaches to respond to clinical variation and understand when action is warranted. Evaluation of the effectiveness of particular feedback approaches is now required to determine if there is an optimal approach to create change where needed.

## Background

Assessment of clinical variation has attracted increasing interest in health systems internationally due to growing awareness about better value and appropriate health care as a mechanism for enhancing efficient, effective and timely care [1–3]. Countries including the United States of America (USA), Canada, Spain, United Kingdom (UK), Germany, the Netherlands, Norway, New Zealand and Australia have produced atlases of variation in health care to guide system and service improvements [4–6]. Through these atlases, substantial variations in the healthcare provided to patients have been identified across each country, with implications for patient outcomes [7]. Variations have been reported in a range of care areas including surgery for hysterectomy, cataract surgery, planned Caesarian section, arthroscopic surgery and potentially preventable hospitalisations for selected conditions [8, 9].

It is widely acknowledged that not all variation is unwarranted and that some variation may in fact be a marker of effective, patient-centred care [10]. Unwarranted clinical variation (UCV) describes “patient care that differs in ways that are not a direct and proportionate response to available evidence; or to the healthcare needs and informed choices of patients.” [7] Understanding variation and what is unwarranted has been identified as important in guiding value-based healthcare [8, 11]. Value-based healthcare has been conceptualised in the US context in terms of the ‘health outcome achieved per dollar spent,’ but more recently in the UK in terms of optimising the value of resources through their utilisation for each patient sub-group, which is determined by clinicians [12, 13]. In healthcare systems such as the US, healthcare providers are also transitioning from volume-based to value-based payments for care. In the context of these shifts, understanding the variations that exist and care that is considered ‘low value’ are critical [11, 12, 14, 15].

Application of well-established statistical frameworks to the processes and treatments undertaken across health systems internationally has produced a substantial body of literature documenting the nature of variations [16–18]. Whilst methods for detecting variation, such as exploring statistically significant deviation from acceptable parameters, are widely acknowledged, methods for determining variation that warrants action or is considered problematic, is debated strongly [18]. Furthermore, the optimal approach for reducing UCV is also unclear. In 2017, a review of approaches to address UCV highlighted that determining clinical variation that is unwarranted is a challenge for care decisions that may vary based on patient preferences or for which there is mixed evidence of its effectiveness [19].

Feedback using administrative databases to provide benchmarking data has been utilised in several countries to explore clinical care variation and to enhance guideline adherent care [18, 19]. The Australian Commission on Safety and Quality in Health Care has developed the Framework for Australian Clinical Quality Registries as a mechanism for governments and health services to capture the appropriateness and effectiveness of care within their jurisdiction [20, 21]. In the UK, clinical registries have been adopted and also linked with financial incentives encouraging appropriate care. Mechanisms for providing immediate feedback to individual clinicians about their practice are also identified in the context of responding to clinical variation, with training and checklists to accompany feedback data [22, 23]. Furthermore, the provision of feedback using these clinical registry data has been identified as an approach that can contribute to improved patient outcomes [24].

An extensive literature has explored the impact of audit and feedback approaches as methods for changing health professional practice, addressing variations and the quality of care, with publications focused to quantitative synthesis [25–27]. The value of feedback approaches to address unwarranted clinical variation across health systems and services, explored through a range of study designs, has not been subject to evidence synthesis. Synthesis is required to explore the range of approaches taken by healthcare teams, services or at a network or system level in using feedback approaches to address unwarranted clinical variation and the data regarding their effectiveness. This review sought to address this knowledge gap by answering the questions below.

### Review questions

What are the feedback approaches currently used to address unwarranted clinical variation and what is the evidence of their effectiveness?

## Method

This literature review utilised a rapid evidence assessment (REA) methodology, which employs the same methods and principles as a systematic review but makes concessions to the breadth or depth of the process to suit a shorter timeframe and address key issues in relation to the topic under investigation [28]. For example, in this case we were establishing evidence relevant for a contemporary policy issue requiring a time-sensitive, evidence-informed response. The review protocol was therefore also not registered. The purpose of a REA is to provide a balanced assessment of what is already known about a specific problem or issue. REAs utilise strategies to assist in facilitating rapid synthesis of information. In this instance, the strategies used were to limit the number of data sources searched to the major databases in the field of healthcare quality improvement [29]. The Preferred Reporting Items for Systematic Reviews and Meta-Analyses—PRISMA statement—was used to guide reporting of this rapid review [30].

### Eligibility criteria

Publications were included if they were available in English, reported original primary empirical or theoretical work, were published from January 2000–August 2018, involved public or private hospitals, day procedure centres, general practice or other primary/community care facilities. Conceptual, theoretical, quantitative or qualitative studies of any research design were included. Studies had to report the use of any mode of feedback to respond to clinical variation, with a focus to addressing unwarranted variations. The definition for facilitated feedback applied in this work was the reporting of outcomes directly to key stakeholders with dialogue geared toward change or any other activities to support change that addressed unwarranted variation. Studies reporting feedback processes provided by health system agencies or directly to health services providers, health districts, or clinicians were eligible. Studies were eligible if they reported feedback in the context of continuous quality improvement, defined as the use of quality “indicators” to initiate and drive practice changes in an ongoing cycle of continuous improvement. Reported outcomes had to include perceived or actual change in clinical practice variation.

### Study identification

A range of text words, synonyms and subject headings were developed for the major concepts of clinical variation, quality improvement and feedback. These text words, synonyms and subject headings were used to undertake a systematic search of two electronic databases that index journals of particular relevance to the review topic (Medline and PubMed) from January 2000 to August 2018 in order to focus the search for contemporary policy development (See Additional file 1 for electronic search strategy). Hand searching of reference lists of published papers ensured that relevant published material was captured. Results were merged using reference-management software (Endnote, version X8) and duplicates removed.

### Study selection and data extraction

Three reviewers (EM, DH, RH) independently screened the titles and abstracts. Copies of the full articles were obtained for those that were potentially relevant. Inclusion criteria were then independently applied to the full text articles by each of the members of the reviewer team (all authors). Disagreements were resolved through final discussion between two members of the review team (RH, EM). The following data were extracted from eligible literature: author(s), publication year, sample, setting, objective, feedback approach and main findings.

### Narrative data synthesis

Findings were analysed using a narrative empirical synthesis in stages, based on the study objectives [28, 31]. A narrative approach was necessary in order to synthesise the qualitative and quantitative findings. A quantitative analytic approach was inappropriate due to the heterogeneity of study designs, contexts, and types of literature included. Initial descriptions of eligible studies and results were tabulated (Appendix). Patterns in the data were explored to identify consistent findings in relation to the study objectives. Interrogation of the findings explored relationships between study characteristics and their findings; the findings of different studies; and the influence of the use of different outcome measures, methods and settings on the resulting data. The literature was then subjected to a quality appraisal process before a narrative synthesis of the findings was produced.

### Assessment of the quality of the studies

An assessment of study quality was undertaken using the Quality Assessment Tool of Studies of Diverse Design (QATSDD) for assessing heterogeneous groups of studies [32]. This tool is suitable for assessing the quality and transparency of reporting of research studies in reviews that synthesise qualitative, quantitative and mixed-methods research. Publications identified in the database search were scored against each criterion on a four-point scale (0–3) to indicate the quality of each publication and the overall body of evidence. The criteria are shown in Table 1.

**Table 1:** Data appraisal items

Quality criteria	
Explicit theoretical framework	
Statement of aims/objective in body of report	
Clear description of research setting	
Evidence of sample size considered in terms of analysis	
Representative sample of reasonable size	
Description of procedure for data collection	
Rationale for choice of data collection tool	
Detailed recruitment data (no. approached, declined etc.)	
Statistical assessment of reliability & validity of measurement tools (quantitative)	
Fit between study objectives & method of data collection	
Fit between study objectives & content of data collection tool	
Fit between study objectives and method of analysis	
Good justification for method of analysis	
Assessment of reliability of analytic process (qualitative)	
Evidence of user involvement in design (e.g. pilot work)	
Strengths & limitations critically discussed	

## Results

### Results of the search

**Fig. 1:**
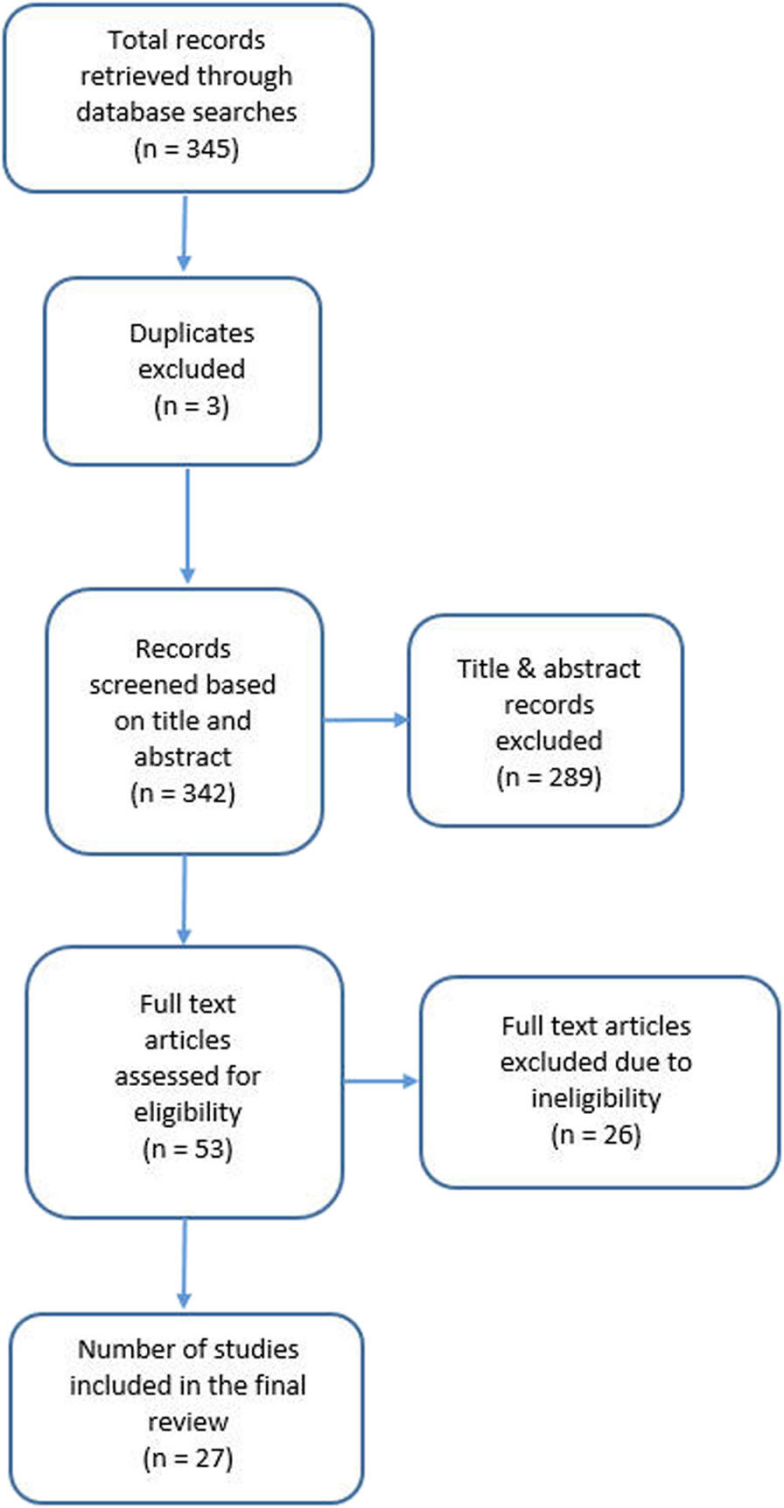
Flow chart of study selection process

After removing duplicates, 342 records were identified. Title and abstract screening review resulted in 53 publications that fulfilled the inclusion criteria (Fig. 1). Twenty-seven studies were included in the review. Feedback approaches that occurred over a duration of 1 month to 9 years to address clinical variation emerged from 27 publications with quantitative (20), theoretical/conceptual/descriptive work (4) and mixed or multi-method studies (3). A summary table of included studies and feedback approaches used is shown in Table 2.

**Table 2:** Summary of included studies

Author	Year	Country	Study design and duration	Setting	Sample	Aim	Feedback approach
Al Mohiza	2016	US	Cluster randomised trial 16 weeks	15 outpatient neurological speciality clinics	23 physical therapists	To implement and evaluate a quality improvement initiative in neurologic outpatient practice.	Process variation Feedback via email of educational material, education via webinar and brief assessment and reminders.
Abdul-Baki	2015	US	Pre and post-study 4 years	1 metropolitan endoscopy centre.	17,526 colonoscopy reports	To assess whether public reporting of colonoscopy quality was associated with improvement in adenoma detection rate.	Reporting of quality measures Feedback via a published public report of each physician’s overall quality of colonoscopy score.
Baker	2008	US	Pre and post-study 4 years	Cardiac Surgery Research database	979 cardio-pulmonary bypass patients	To demonstrate the influence of automated generation of quality indicators for cardiopulmonary bypass and the implementation of a CQI program on the process of care.	Local QI feedback Feedback of data generated in an electronic report was discussed in small groups using QI methods for change.
Caterson	2015	US	Methodological work	1 tertiary hospital	Impact-based reconstruction	To investigate the standardised clinical assessment and management plan concept for breast reconstruction.	Guideline Feedback through local data coordinators to ensure physicians via their performance and record their decision-making.
Cook	2014	US	Pre and post-study 2 months	1 hospital progressive care unit	86 baseline and 187 intervention surgical patients	To improve the quality of care in indwelling catheter use following surgery.	Health information technology -Decision support Rapid recovery pathway provided triggers for catheter removal. Educational reinforcement of the process provided to the team.
Das	2008	UK	Between groups survey 1 year	British Society of Gastroenterology Membership	228 gastroenterologists	To provide a review of the management of Barretts Esophagus in the UK and compare to national guidelines.	Reporting of quality measures The process of being in the AspECT trial in which sites received data of their practice was feedback of their practice.
Dorfsman	2018	US	Pre and post-study 1 year	3 emergency medicine programs in academic health centres	31 residents	To use clinical practice variations as a training tool for residents.	Process variation Feedback via resident educational small group discussion sessions reviewing their practice.
Deyo	2000	US	Pre and post-study 1 year	22 health organisations including 12 hospitals, insurance plans, multicentred health services and independent services.	3 team members from each of the 22 organisations	To use scientific evidence and behaviour change approaches to improve care for back pain.	Process variation Feedback through quarterly meetings reviewing each organisations data and group coaching sessions for each organisation.
Dykes	2005	US	Pre and post-study 1 year	1 community hospital	Pre-test sample - 90 heart failure patients over 65 and 55 control stroke patients over 65. Post-test sample - 96 heart failure patients over 65 and 75 control stroke patients over 65	To examine interdisciplinary knowledge and adherence to core recommendations before and after HEART Failure Effectiveness and Leadership Team intervention.	Local QI feedback Multifaceted feedback approaches including small group discussion of practice data and information provision regarding best practice adapted to local context.
Eagar	2010	Australia	Conceptual 18 months of data collection.	Palliative Care Outcomes Collaboration of 111 services	Benchmarking round 1–51 services; Benchmarking round 2–94 services	To measure the outcomes and quality of specialist palliative care services and to benchmark services on a national basis through an independent third party.	Reporting of quality measures Feedback on nationally reported data supported by quality improvement facilitators working with each service.
Fredriksson	2017	Sweden	Cross sectional survey 1 month	78 hospitals reporting to The Swedish Registry of Gallstone Surgery and Endoscopic Retrograde Cholangiopancreatography, 71 hospital clinics reporting to the Swedish Stroke Register and 31 hospital clinics reporting to the Swedish Lung Cancer Registry	3–6 respondents from each organisation	To investigate the use of national quality registries in local quality improvement.	Reporting of quality measures Quality data available to organisations used in various ways by each for QI locally.
Gaumer	2008	Egypt	Case study	14 primary care clinics	NA	To develop a health information system to support quality improvement approaches to help clinicians understand practice variation.	Local QI feedback Feedback through a purpose-built electronic report on individual practice.
Grey	2014	New Zealand	Cross sectional survey	Public and private health sector organisations in New Zealand	28 stakeholders one-on-one feedback and 100+ meeting attendees	To gain feedback about the interpretation and use of Atlas data for frontline quality improvement.	Reporting of quality measures One to one feedback and multiple group feedback and discussion events.
Griffiths	2017	UK	Pre and post-study 7 months	Royal College of Pathologists	Training event and grand rounds resulted in 50 checklists completed - no data re attendance of these.	To investigate the feasibility of developing key performance indicators to measure adherence to a specified process of histopathological surgical dissection.	Local QI feedback Key performance indicators used to create checklists to reduce UCV. Training events held on the checklists.
Ip	2014	US	Pre and post-study 21 months	183 practices within an integrated health system	2240 adult lower back pain patients between 2007 and 2010	To examine the impact of a multi-faceted clinical decision support intervention on MRI use in patients with lower back pain.	Health information technology -Decision support Feedback through peer to peer consultation and electronic practice variation reports provided to clinicians.
Kelly	2016	Australia	Pre and post-study with qualitative interview 1 year	48 hospitals	149,888 patients undergoing percutaneous coronary intervention 2002–2004	To demonstrate that meaningful interpretation from funnel plots can be derived from a New York dataset.	Local QI feedback Awareness activities and educational sessions.
Lee	2016	US	Pre and post-study 3 years	Community and specialist inflammatory bowel disease clinics in one health service	50 electronic medical charts of 6 gastroenterology fellows	To incorporate an in-service educational session on IBD health maintenance to increase trainees’ knowledge and awareness.	Process variation Feedback through in-service educational sessions.
McFadyen	2015	Canada	Pre and post-study 14 months	One provincial health region	56 clinicians - general surgeons, surgical oncologists, urologists and pathologists.	To provide clinicians with an individualised feedback report to improve quality.	Local QI feedback Confidential individual electronic provider reports of their practice data.
Miller	2011	US	Pre and post-study 16 months	Three urology practices.	858 urology presentations	To improve patterns of care for radiological staging of newly diagnosed prostate cancer.	Local QI feedback Feedback activities included review of data against clinical guidelines, local meetings and collaborative-wide teleconference discussions.
Min	2017	Canada	Pre and post-study 3 years	One major acute care centre.	43 emergency physicians	To determine whether point of care clinical decision support can effectively reduce inappropriate medical imaging of patients who present to the emergency room with low back pain.	Health information technology - Decision support Checklist in the computerised order entry system developed by working group of clinicians and embedded to prompt practice change.
Nguyen	2007	US	Pre and post-study 4 years	44 facilities in the Northwest Renal Network	4 workshops attended by - 36 nephrologists, 16 VA surgeons and 1 radiologist; 35 physicians responded to the follow-up survey	To use educational interventions to promote arteriovenous fistula creation.	Process variation Workshop meetings across the network.
Nordstrom	2016	US	Pre and post-study 9 months	Cohorts of physician practices across Vermont	28 physician practices in 4 cohorts	To examine physician engagement and change in buprenorphine practice	Process variation Learning collaborations of face to face and teleconference sessions.
Rubin	2015	UK	Mixed methods 4 years	8179 primary care practices	92 interviewees - GP, GP cancer leads, public health staff and cancer network staff.	To explore whether quality improvement activities were associated with a change in referral practice.	Local QI feedback GP leads in cancer networks across the country used different QI approaches.
Smith	2013	Australia	Pre and post-study 9 years 3 months	Cardiac surgical unit at one hospital	5265 consecutive cardiac procedures 2003–2012	To explore the application of graphical statistical process techniques to inform routine cardiac surgical mortality and morbidity review processes.	Local QI feedback Group reviews of control charts and funnel plots.
Stafford	2003	US	Pre and post-study 9 months	117 primary care providers associated with one hospital	105,682 patients and 511,328 patient visits	To evaluate the impact of a feedback intervention on reducing rate and variation of ECG orders.	Local QI feedback Quality improvement team at each hospital presented to clinicians their performance data.
Tavender	2015	UK	Conceptual	One emergency department	NA	To develop a targeted theory-based intervention that improves the management of mild traumatic brain injury.	Local QI feedback Behaviour change techniques from the theoretical domains framework.
Tomson	2013	The Netherlands	Narrative review	NA	NA	To describe quality improvement techniques that maintain clinical quality.	Local QI feedback Multiple approaches discussed through included articles.

#### Excluded studies

Studies were excluded at the full-text review stage because they did not meet the inclusion criteria in being primary empirical or theoretical work (*n* = 17) or did not include a feedback approach (9).

#### Study quality

The data appraisal identified that the studies reported in included papers were generally of good quality with particular strengths in the use of evidence-based quality improvement strategies, selection of appropriate study designs, and application of rigorous analytic techniques. A key limitation across the body of evidence was the use of a small sample, often in a single site study, limiting the generalisability of results.

### Review findings

The included studies were reported from nine countries: US (14), UK (4), Australia (3), The Netherlands (1), Canada (2), Sweden (1), Egypt (1), and New Zealand (1).

#### National reporting and feedback

Four studies outlined approaches for benchmarking care nationally or in contributing to publicly-reported datasets as strategies to identify variation that may be problematic, and to incite change [33–36]. These studies incorporated steps to address variation by providing feedback to service providers about the variations arising in their care compared to benchmarks. Eagar et al. 2010 reported the Palliative Care Outcomes Collaboration (PCOC) to measure the outcomes and quality of palliative care services and to benchmark across Australia. A PCOC quality improvement facilitator met with the services in the collaboration to embed the collection of standardised clinical assessment into practice to improve care quality, in addition to convening national benchmarking meetings. The success of the approach at reducing variation or addressing unwanted variation was not reported [35].

The role of national quality registries in quality improvement was explored in one study [33]. The authors explored the use of quality registry data amongst heads of clinics and clinicians in quality improvement activities as a strategy to address variation. The findings indicate that national quality registries can provide data that, when used in feedback to staff, can provide the basis for identifying and discussing variations and appropriate responses. Use of national quality registries varies widely and these are not routinely incorporated into efforts to address variation [33]. Similarly, Grey et al. (2014) explored how the Atlas of Healthcare Variation in New Zealand is presented, interpreted and applied as a tool to understand and target variation within a quality improvement paradigm. Stakeholders reported using funnel plots to enable clinicians to benchmark against peers and identify areas of variation for scrutiny. This benchmarking provides the basis for quality improvement activities to address variation [36]. The study by Abdul-Baki et al. (2014) reported that public reporting as an intervention was associated with an increase in adenoma detection rates in a private endoscopy practice. The investigators of this study suggested that simply providing feedback data may improve care quality and reduce variations [34]. However, the mechanism by which this feedback approach may work is not established and the pre- and post-study design used was not sufficiently sensitive or controlled to determine causation. On a smaller scale, in a secondary analysis of 228 senior gastroenterologists, Das et al. (2008) reported that data on the quality and management of Barrett’s esophagus (BE) through surveillance also led to reduced variation from the adherence to the recommended four-quadrant biopsy protocol for histological sampling of those with macroscopically suspected BE [37].

#### Local reporting and feedback

Data were captured about the practice of individuals or teams and reported back at local level within a network, an organisation, an organisational unit or to individuals in six studies [38–43]. Individual provider reports were explored in two studies [38, 39]. In a study by Stafford (2002), primary care providers were given data over a nine-month period comparing their use of the electrocardiogram (ECG) compared to peers to reduce non-essential ECG ordering based on a range of national guidelines and recommendations. Variation in the ordering of ECG and its use reduced after the nine-month period [39]. In a project exploring variation in two pathology indicators: one for prostate and one for colorectal cancer, urologists, surgeons and pathologists of four hospitals were provided data supported by evidence-based guidelines [38]. The aim was to encourage behaviour change and improving quality through reduced unwarranted variation. Individual feedback increased appropriate treatment demonstrated in a reduced prostate margin positivity rate from 57.1 to 27.5% on one indicator but did not impact the colorectal cancer indicator [38]. A key finding was the group of urological surgeons who did not show improvement on one of the indicators also had the poorest attendance at the engagement sessions held before and during the project [38].

Chart review was used in a study by Kelly et al. (2016) to establish adherence to the local treatment pathway for the management of atrial fibrillation with rapid ventricular response (AFRVR). Local teams made emergency departments aware of their adherence levels and best practice guidelines leading to a substantial increase in adherence to the pathway from 8 to 68% over the nine-month period [40]. Qualitative findings revealed success factors to be a strong local clinical lead with multi-disciplinary team support, access to evidence-based resource materials, regular feedback about performance throughout the process [40].

Local monitoring and feedback were also used by Smith et al. (2012) to review and understand variation in cardiac surgical procedures. Data from the regular monitoring of quality data between 2003 and 2012 was reported back to the cardiac surgery unit’s bi-monthly morbidity and mortality meetings in order to explore variations and determine action to be taken. The authors reported that this approach was valuable in distinguishing individual and systemic variation issues and those requiring action [41].

In primary care, Gaumer, Hassan and Murphy (2008) developed an information system, ‘Feedback and Analytic Comparison Tool’ to enable clinicians to monitor their own performance data and act accordingly. This system provided feedback to allow clinicians to identify practice variations but did not utilise health information technology (HIT) to identify feedback warranting action [42].

One study explored provision of data across a network [43]. A cancer primary care network in the UK identified a clinical audit and the provision of risk assessment tools as two of four quality improvement approaches for reducing variation. The impact of clinical audit feedback alone was not established in isolation of the other quality improvement activities but a significant increase of 29% in referral rates was reported across the participating general practices [43]. In the context of cancer networks, clinicians felt better supported to sustain improvement efforts to address UCV when there was effective leadership marked by organisational stability and consistent messaging [43].

#### Facilitated feedback

Fifteen studies employed facilitated feedback methods to explore variations and address areas in which changes were required. The largest group of facilitated feedback approaches were identified in local-level small scale quality improvement projects within health services (3), or those that operated across an organisation (2) or network (6). One paper was a review of multiple quality improvement projects [44]. HIT was identified in several studies as part of the approach to identifying variation, but a sub-set of three studies focused on HIT methods for providing facilitated feedback on variation warranting action.

##### Quality improvement projects

Twelve quality improvement (QI) projects were retrieved from the search, most of which identified process variation and then utilised educational approaches to change clinician behaviour [23, 24, 45–53]. Table 3 provides a summary of the projects identified. Approaches taken to inform the facilitated feedback approaches included the use of the Theoretical Domains Framework for behaviour change, clinical algorithms as a basis for understanding variation and HIT for implementation [45, 47–49]. In their narrative review, Tomson and Sabine (2013) detail a range of local and national projects in the UK that utilise evidence-based guidelines to support QI initiatives to address unwarranted variation. They reported that local level QI projects that engaged a package of clinical actions to achieve the improvement aim were those that saw reductions in problematic variation and enhanced quality. The authors also highlight the inefficiency of a multitude of local level projects and the potential value but also challenges of national or collaborative approaches. A central difficulty identified by the review authors was the completion of such QI initiatives as an additional activity to routine clinical work [44]. This finding was reflected in several of the included studies.

**Table 3:** Summary of quality improvement projects

Author	Date	Country	Setting	Sample	Summary
Almohiza	2016	US	15 outpatient neurological speciality clinics	23 physical therapists	Reported a 16-week quality improvement project amongst physical therapists working in rehabilitation services in the US. A clinical treatment algorithm was developed to determine evidence-based effective practices and deviation from these was considered ‘non-compliant’, indicating problematic variation. Following a behavioural intervention program including a webinar, test and competency training, adherence to the processes identified as effective by the clinical algorithm was assessed and improved by 5–10%. Over utilised treatments reduced by 16% post-intervention.
Baker	2008	US	Cardiac Surgery Research database	979 cardio-pulmonary bypass patients	Reported findings of a project to reduce variation in the care process for cardiac surgical patients that compared no QI data with automated QI data alone and automated QI data with implementation of a continuous quality improvement project. This study pulls together the use of health information technology, quality reporting and improvement interventions. Adherence to protocol and reduction in practice variation was enhanced in the automated feedback programme but optimised by the use of a CQI approach.
Caterson	2015	US	1 tertiary hospital	Impact-based reconstruction (methodological work)	Reported the development and use of a Standardised Clinical Assessment and Management Plan (SCAMP) in plastic surgery with a decision-tree algorithm. Adherence to the SCAMP algorithm was used to identify variation and direct quality improvement efforts to address this.
Deyo	2000	US	22 health organisations including 12 hospitals, insurance plans, multicentred health services and independent services.	3 team members from each of the 22 organisations	Measurement and education program with 22 participating organisations including health plans and medical centres. Those organisations and service with “outlier” rates of imaging or referral (identified as statistical outliers from the normal range of imaging or referral in each organisation) were used to identify clinics or physicians for targeted intervention. The intervention program including three learning sessions, focusing on areas of practice variation identified by the participating organisations from their own data, in addition to a final national congress. Participants worked within their own teams to problem-solve and then across teams from other organisations. A key component of the process was to present their clinical variation data and perform continuous repeated measurements to track change in variations. Findings suggest that the approach was effective in reducing unwarranted variations, although outcome measures used to assess variation were different across the participating sites based on their clinical goals and data sources. Reduced variations were identified in outcomes such as levels of x-rays ordered, prescribed bed-rest and also increased the use of patient education materials by 100% that may also work to address unwarranted variations
Dorfsman	2018	US	3 emergency medicine programs in academic health centres	31 residents	Utilised variations from guideline-based care in the organisation’s emergency medicine to develop educational sessions for residents working in that department on a monthly basis. The sessions explored the evidence base for a particular practice and variation, expert discussions on areas in which the evidence base was not conclusive regarding effective care and encouraged debates between residents attending [54]. Findings did not establish whether the training addressed unwarranted variations or changed behaviour, but 77% of the 31 residents surveyed indicated that the sessions aided their understanding of why clinical practice variations may occur
Dykes	2005	US	1 community hospital	Pre-test sample - 90 heart failure patients over 65 and 55 control stroke patients over 65. Post-test sample - 96 heart failure patients over 65 and 75 control stroke patients over 65	Incorporated an automated care pathway in the electronic medical record into an intervention that included evidence provision to clinicians and patients, a self-management tool and discipline-specific feedback regarding guideline adherence to enhance care for stroke patients. The study reported that point of care evidence enhances adherence to guidelines including those around patient self-management education in stroke care.
Griffiths	2017	UK	Royal College of Pathologists	Training event and grand rounds resulted in 50 checklists completed - no data re attendance of these.	Key performance indicators were used to identify variations in individual practice and report this back alongside a quality improvement project. The project included implementing four checklists based on evidence-based guidelines along with a weekly training event to try to reduce variations in pathology practices. The project isolated the effect of the intervention from the training component and established that utilising a checklist alone was associated with conforming to the evidence-based approach rather than the addition of the training component. Having the checklist available at the point of dissection was critical.
Lee	2016	US	Community and specialist inflammatory bowel disease clinics in one health service	50 electronic medical charts of 6 gastroenterology fellows	A random selection of medical records was audited against 15 quality measures for inflammatory bowel disease and then re-audited after an educational session in which the quality measures and performance against these was reviewed. Lee at al identified a positive correlation between the intervention and compliance with the quality measures, with compliance increasing by 16%
Miller	2011	US	Three urology practices	858 urology presentations	Between 2009 and 2010, Urological Surgery Quality Collaborative surgeons collected data for men with newly diagnosed prostate cancer through 3 phases of data collection. In phases 2 and 3, collaborative quality improvement interventions, including comparative performance feedback, and review and dissemination of clinical guidelines were used. The use of bone scans and computerized tomography across prostate cancer risk strata, Urological Surgery Quality Collaborative practice locations, and before and after quality improvement interventions was examined.
Nguyen	2007	US	44 facilities in the Northwest Renal Network	4 workshops attended by - 36 nephrologists, 16 VA surgeons and 1 radiologist; 35 physicians responded to the follow-up survey	A network education model was reported as a strategy to reduce unwarranted variation in dialysis using arteriovenous fistula (AVF). Forty-six facilities contributed to four targeted regional workshops that explored the root causes of low AVF rates by interviews with vascular surgeons, nephrologists, dialysis staff, and interventional radiologists. The analysis identified three key barriers to a higher AVF rate: 1) Failure of nephrologists to act as vascular access team leaders; 2) Lack of AVF training for vascular access surgeons, including vessel assessment skills, vein mapping, and complex surgical techniques and 3) Late referral of chronic kidney failure (CKF) patients to nephrology. A literature review was then conducted to identify best demonstrated practice regionally and the strategies successfully used by this team were included in the quality improvement project. Four intervention workshop meetings were held and intervention site participants took away follow-up materials to address the content locally. Of the 35 attending physicians, 91% reported that they had changed their practice to address variations based on the intervention in consistent areas relating to AVF use over the five-year period in which outcome data were collected
Nordstrom	2016	US	Cohorts of physician practices across Vermont	28 physician practices in 4 cohorts	Reported the impacts of a learning collaborative between 28 physician practices that collected and reported on their quality improvement data through four sessions, in addition to didactic lectures, case presentations and discussion of practice-improvement strategies to reduce variation in the provision of buprenorphine. Findings indicated that there was a substantial reduction of up to 50% in variations across all seven quality measures. A collaborative in urological surgery adopted a facilitated feedback approach with performance feedback and review in relation to clinical guidelines [50]. The authors reported that the urological collaborative demonstrated substantial reductions in variations in practice patterns and guideline adherence following the feedback intervention
Tavender	2015	UK	One emergency department	Theoretical work	Described the process of applying two theoretical frameworks to investigate the factors influencing behaviour and the choice of behaviour change techniques. Two theoretical frameworks were used together to inform intervention development in managing mild traumatic brain injury in the ED. The intervention approach included a range of modes to encourage optimal behaviours in care delivery for managing mild brain trauma.
Tomson	2013	The Netherlands	Review	Review	Local level QI projects that engaged a package of clinical actions to achieve the improvement aim were those that saw reductions in problematic variation and enhanced quality. The authors also highlight the inefficiency of a multitude of local level projects and the potential value but also challenges of national or collaborative approaches. A central difficulty identified in this review is the completion of such QI initiatives as an additional activity to routine clinical work.

At the simplest level, Lee at al (2016) reported a process in which a random selection of medical records was audited against 15 quality measures for inflammatory bowel disease. They then re-audited after an educational session in which performance against the quality measures was reviewed. Lee at al identified a positive correlation between the intervention and compliance with the quality measures, with compliance increasing by 16% [53]. Two studies progressed this approach by developing algorithms for a range of evidence-based practices as the basis for determining compliance [45, 47]. Key performance indicators were used by Griffiths and Gillibrand (2017) to identify variations in individual practice and report this back alongside a quality improvement project [24]. The project included implementing four checklists based on evidence-based guidelines along with a weekly training event to try to reduce variations in pathology practices. The project isolated the effect of the intervention from the training component and established that utilising a checklist alone was associated with conforming to the evidence-based approach rather than the addition of the training component [24].

At a network level, a measurement and education project was reported by Deyo et al. (2000) with the Institute for Healthcare Improvement to address variations in lower back pain care across 22 participating organisations including health plans and medical centres. Those organisations and service with “outlier” rates of imaging or referral (identified as statistical outliers from the normal range of imaging or referral in each organisation) were used to identify clinics or clinicians for targeted intervention [49]. The intervention program including three learning sessions, focusing on areas of practice variation identified by the participating organisations from their own data, in addition to a final national congress. Participants worked within their own teams to problem-solve and then across teams from other organisations. A key component of the process was to present their clinical variation data and perform continuous repeated measurements to track change in variations. Findings suggest that the approach was effective in reducing unwarranted variations, although outcome measures used to assess variation were different across the participating sites based on their clinical goals and data sources. Reduced variations were identified in outcomes such as levels of x-rays ordered, prescribed bed-rest and also increased the use of patient education materials by 100% that may also work to address unwarranted variations [49].

A further network education model was reported by Nguyen et al. (2007) as a strategy to reduce unwarranted variation in dialysis using arteriovenous fistula (AVF) [51]. Forty-six facilities contributed to four targeted regional workshops that explored the root causes of low AVF rates by interviews with vascular surgeons, nephrologists, dialysis staff, and interventional radiologists. The analysis identified three key barriers to a higher AVF rate: 1) Failure of nephrologists to act as vascular access team leaders; 2) Lack of AVF training for vascular access surgeons, including vessel assessment skills, vein mapping, and complex surgical techniques and 3) Late referral of chronic kidney failure (CKF) patients to nephrology. A literature review was then conducted to identify best demonstrated practice regionally and the strategies successfully used by this team were included in the quality improvement project. Four intervention workshop meetings were held and intervention site participants took away follow-up materials to address the content locally. Of the 35 attending physicians, 91% reported that they had changed their practice to address variations based on the intervention in consistent areas relating to AVF use over the five-year period in which outcome data were collected [51]. Similarly, Nordstrom et al. 2016 report the impacts of a learning collaborative between 28 primary care practices that collected and reported on their quality improvement data through four sessions, in addition to didactic lectures, case presentations and discussion of practice-improvement strategies to reduce variation in the provision of buprenorphine [52]. Findings indicated that there was a substantial reduction of up to 50% in variations across all seven quality measures [52].

##### Health information technology (HIT)

Progressing a thread within many quality improvement projects that were reported, three studies outlined HIT clinical decision support tools explicitly as tailored feedback approaches to reduce unwarranted variation. Two studies reported clinical decision support tools to optimise the appropriate use of imaging for lower back pain [55, 56]. Ip et al. (2014) report a clinical decision support intervention on magnetic resonance imaging (MRI) for low back pain, which incorporated two accountability tools. One component of the intervention was a mandatory peer-to-peer consultation when test utility was uncertain. The second intervention component was quarterly practice variation reports to providers. The multi-faceted intervention demonstrated a 32–33% decrease in the use of MRI for any body part, indicating that this approach could address unwarranted variation relating to overutilisation [55]. Min et al. (2017) embedded a point of care checklist in the computerised entry form for image ordering in addition to a patient education program in which a summary document explaining when medical imaging is necessary was included in the lower back pain pamphlet [56]. Post-intervention, the median proportion of lower back pain patients who received an imaging order reduced by 5% and the median decrease of image ordering amongst the 43 emergency department physicians in the study reduced by 13% [56].

Cook et al. (2014) utilised HIT to develop a mechanism for determining pre-operatively those patients for whom a standardised care pathway would be appropriate in their cardiac surgical care [54]. Post-operatively, the patients on the standardised pathway are confirmed as continuing this pathway in Intensive Care Unit (ICU) and then to the Progressive Care Unit. For those remaining on the pathway, an electronic protocol triggers the removal of the bladder catheter; therefore, practice variation in the time to remove a catheter for those on the pathway should be minimal. The electronic decision tool was complemented by quality improvement methods including educational reinforcement and procedural training around catheter removal, and performance reports provided back to staff at 1, 3 and 6-month intervals. Findings indicated that an improvement from 91% at baseline to 97% post-intervention compliance with guidelines was achieved in relation to removal of catheter, suggesting that the decision support tool contributed to reduced unwarranted variation [54].

## Discussion

Responses to clinical variation range from simply presenting evidence to individuals, teams and organisations, to facilitated tailored feedback that may be integrated in broader quality improvement projects. Whilst providing feedback on clinical variation data alone can encourage reflection and improvement, data tailored to particular health professionals, services or systems, and disseminating information to these audiences via facilitated feedback processes, may have greater capacity to drive large-scale change. Current evidence demonstrates variability in approaches to providing feedback around variation. No single optimal model for structuring facilitated feedback was identified as widely adopted. Insufficient evidence was available to determine that one feedback approach is more or less effective than another.

Extensive theory-based research in the psycho-social literature has provided evidence of the critical elements of feedback that influence behaviour change, including aspects of the content and delivery of feedback [57]. Yet, as evident in the quality appraisal process, the included studies rarely referred to any theoretical basis for the interventional approach in the context of addressing UCV. It is apparent that many non-experimental approaches used to provide clinician feedback at the clinical front-line, system and service level are not grounded in theory, which creates challenges for understanding how and why an approach or its elements did or did not work to address UCV. Although theory-based approaches were not explicitly identified, it is apparent that features of the interventions identified in this review reflect common behaviour change techniques, such as the use of goal-setting, self-monitoring and prompting. Further integration of theory into practice would be valuable in the context of addressing UCV to understand the mechanisms by which feedback approaches may or may not work and how these may be utilised across teams, services and systems [58].

Most approaches identified for responding to variation and reducing unwanted variation focus solely or predominantly on variations in clinicians’ practice over a period of several months to several years [19]. This type of variation is important to tackle, but the limited scope of work does not give sufficient consideration to variation due to patient preferences or factors [59]. Mercuri and Gafni (2017) highlight a range of evidence that indicates only around 5–10% of variations relate to clinician choice [59]. There is a need to more fully understand the roles of patient preference and factors in variation, which is information that can be captured and integrated in facilitated feedback approaches. Studies that examined the impact of decisions based on deviations from guidelines (e.g. limiting MRI ordering rights for GPs) in terms of cost and care improvements were lacking. This information is important when considering UCV as a system-wide concept.

### Implications

HIT was the principal method for capturing and, in some cases, reporting variation data back to facilitate change [54–56]. HIT was central to continuous quality improvement projects that occurred in teams or organisations, for example through generation of clinical treatment algorithms and automated generation of quality indicators to drive or contribute to the feedback sessions [46]. Outcomes that were assessed in facilitated feedback and enabled continuous quality improvement approaches included reduced overuse of technologies or treatments, changes in patient clinical outcomes and adherence to practice protocols [54–56].The increasing availability of HIT and real-time analytics in health services internationally makes it likely that the relationship between HIT and clinical variation data and subsequent behaviour change will only continue to strengthen over time. There are opportunities for exploring the use of HIT in recording patient preferences as a feedback approach to contribute to understanding and reducing unwarranted variations.

In the context of uncertainty regarding how to define and tackle unwarranted clinical variation, feedback and clinical review are important as avenues to ensure a nuanced approach. Methods for providing feedback specifically for the purpose of reducing UCV vary between teams, units and organisations. Understanding the features of feedback approaches that are effective in the identification and reduction of UCV is required to support system-wide efforts. This knowledge is crucial for developing an evidence-based methodology to address UCV.

### Limitations

Limiting the electronic search to published works identified in only two databases studies post 2008 may have shaped the review findings. A full-scale systematic review was beyond the scope of the review, which used REA methodology to undertake a focused review to address a contemporary, high priority policy question in Australia and internationally. The breadth of areas covered by the concept of clinical variation would also limit the suitability of a full-scale systematic review to this area.

## Conclusion

Providing feedback to clinicians is identified in a range of settings as being associated with changes in variation such as reducing overuse of tests and treatments, reducing variations in optimal patient clinical outcomes, and increasing guideline or protocol adherence. Feedback approaches that relate to performance indicators may address variations arising due to clinicians’ behaviours but may not necessarily address variations that relate to patient preferences. Evaluation of the effectiveness of approaches utilising facilitated feedback is needed to provide evidence firstly regarding whether facilitated feedback offers advantages over feedback without facilitation in the context of addressing variation, and secondly, to determine if there is an optimal approach to and/or method of facilitation that is more likely to create change where needed.

**Table 4:** Ovid Medline search strategy (run 28/08/18)

#	Searches	Results
1	Practice Patterns, Physicians’/	52989
2	exp physicians/ or clinician*.af. or physician*.af. or exp medical staff/	839680
3	exp hospitals/ or Hospitalization/ or hospitali*.mp.	502959
4	(variation* adj2 (Clinical care or Medical care or Healthcare or health care or Medical practice or physician* or clinical or practice or clinician* or pattern*)).mp.	10132
5	Guideline Adherence/ or Practice Guidelines as Topic/ or Healthcare Disparities/ or clinical protocols/ or organizational policy/ or evidence based*.ti,ab,kw,sh. or exp “Quality of Health Care”/	6335879
6	1 or 2 or 3 or 5	7015351
7	6 and 4	5392
8	6 and ((Regional adj2 variation*) or (geographical adj2 variation*)).mp.	4745
9	7 or 8	9960
10	6 and (small area analysis or small area variation).mp.	1202
11	4 and (regional or geographical).mp.	773
12	exp child/ or exp infant/ or (pediatric* or paediatric* or childhood or children).af.	3005698
13	9 or 10 or 11	11354
14	limit 13 to yr="2000 –Current”	9076
15	limit 14 to english language	8767
16	15 not 12	6914
17	remove duplicates from 16	4015
18	17 and feedback.mp. [mp=title, abstract, original title, name of substance word, subject heading word, floating sub-heading word, keyword heading word, protocol supplementary concept word, rare disease supplementary concept word, unique identifier, synonyms]	67
19	17 and facilitated.mp. [mp=title, abstract, original title, name of substance word, subject heading word, floating sub-heading word, keyword heading word, protocol supplementary concept word, rare disease supplementary concept word, unique identifier, synonyms]	18
20	17 and multifaceted.mp. [mp=title, abstract, original title, name of substance word, subject heading word, floating sub-heading word, keyword heading word, protocol supplementary concept word, rare disease supplementary concept word, unique identifier, synonyms]	18
21	17 and comparative performance.mp. [mp=title, abstract, original title, name of substance word, subject heading word, floating sub-heading word, keyword heading word, protocol supplementary concept word, rare disease supplementary concept word, unique identifier, synonyms]	1
22	17 and “controlled before after studies”.mp. [mp=title, abstract, original title, name of substance word, subject heading word, floating sub-heading word, keyword heading word, protocol supplementary concept word, rare disease supplementary concept word, unique identifier, synonyms]	3
23	17 and ((colleague* or peer*) adj3 assess*).mp. [mp=title, abstract, original title, name of substance word, subject heading word, floating sub-heading word, keyword heading word, protocol supplementary concept word, rare disease supplementary concept word, unique identifier, synonyms]	2
24	17 and (workplace based or work place based or work based).mp. [mp=title, abstract, original title, name of substance word, subject heading word, floating sub-heading word, keyword heading word, protocol supplementary concept word, rare disease supplementary concept word, unique identifier, synonyms]	4
25	17 and facilitator.mp. [mp=title, abstract, original title, name of substance word, subject heading word, floating sub-heading word, keyword heading word, protocol supplementary concept word, rare disease supplementary concept word, unique identifier, synonyms]	4
26	17 and quality improvement.af.	205
27	17 and practice improvement.mp. [mp=title, abstract, original title, name of substance word, subject heading word, floating sub-heading word, keyword heading word, protocol supplementary concept word, rare disease supplementary concept word, unique identifier, synonyms]	8
28	17 and (practice adj2 improvement*).mp. [mp=title, abstract, original title, name of substance word, subject heading word, floating sub-heading word, keyword heading word, protocol supplementary concept word, rare disease supplementary concept word, unique identifier, synonyms]	16
29	17 and evaluation program*.af.	3
30	17 and mentor*.mp. [mp=title, abstract, original title, name of substance word, subject heading word, floating sub-heading word, keyword heading word, protocol supplementary concept word, rare disease supplementary concept word, unique identifier, synonyms]	5
31	17 and continuous quality.mp. [mp=title, abstract, original title, name of substance word, subject heading word, floating sub-heading word, keyword heading word, protocol supplementary concept word, rare disease supplementary concept word, unique identifier, synonyms]	9
32	17 and continuous improvement.mp. [mp=title, abstract, original title, name of substance word, subject heading word, floating sub-heading word, keyword heading word, protocol supplementary concept word, rare disease supplementary concept word, unique identifier, synonyms]	0
33	17 and (quality management or TQM).mp. [mp=title, abstract, original title, name of substance word, subject heading word, floating sub-heading word, keyword heading word, protocol supplementary concept word, rare disease supplementary concept word, unique identifier, synonyms]	41
34	17 and cooperative behavior.af.	24
35	17 and professional development.mp. [mp=title, abstract, original title, name of substance word, subject heading word, floating sub-heading word, keyword heading word, protocol supplementary concept word, rare disease supplementary concept word, unique identifier, synonyms]	6
36	or/18-35	339

## Data Availability

The datasets used and/or analysed during the current study are available in the published works included in the manuscript or from the corresponding author on reasonable request.

## Abbreviations

AFRVR: Atrial fibrillation with rapid ventricular response
AVF: Arteriovenous fistula
ECG: Electrocardiogram
GP: General Practitioner
HIT: Health information technology
ICU: Intensive Care Unit
PCOC: Palliative Care Outcomes Collaboration PRISMA Preferred reporting items for systematic reviews and meta-analyses
QATSDD: Quality Assessment Tool for Studies of diverse design
REA: Rapid evidence appraisal
UCV: Unwarranted clinical variation
UK: United Kingdom
USA: United States of America
